# Sex-Specific Heart Rate Variability Associations With Vitamin B12, Folate, and Iron Status

**DOI:** 10.31083/RCM50065

**Published:** 2026-03-19

**Authors:** Mehmet ÖZYAŞAR, Selçuk ÖZTÜRK, Tolga MEMİOĞLU, Mehmet İNANIR

**Affiliations:** ^1^Cardiology Department, Konya City Hospital, 42020 Konya, Türkiye; ^2^Department of Cardiology, Faculty of Medicine, Izzet Baysal Training and Research Hospital, Bolu Abant Izzet Baysal University, 14280 Bolu, Türkiye

**Keywords:** autonomic nervous system, ambulatory electrocardiography, sex factors, heart rate, iron, folic acid, Vitamin B12

## Abstract

**Background:**

The sex-specific impact of micronutrient status on heart rate variability (HRV) in adults presenting with palpitations to cardiology outpatient clinics remains unclear. Thus, this study aimed to assess the demographic and biochemical determinants of HRV in a clinical cohort of patients presenting with complaints of palpitations.

**Methods:**

This retrospective study included 213 adults aged 18–65 years who presented with palpitations and underwent 24-hour Holter monitoring at our institution between 2023 and 2024. Patients with cardiovascular disease, known arrhythmias, chronic inflammatory conditions, renal dysfunction, or use of medications that affected autonomic function were excluded from the study. Demographic variables, laboratory parameters, and HRV indices were statistically analyzed. The standard deviation of all normal-to-normal intervals (SDNN) was the primary HRV parameter used in both univariate and multivariate linear regression analyses.

**Results:**

The SDNN was significantly lower in women and older adults. In the univariate analyses, age (*β* = –0.203; *p* = 0.003), male sex (*β* = 0.529; *p* < 0.001), ferritin, serum iron, folate, and Vitamin B12 were all associated with the SDNN. However, in the multivariable model, only male sex (*β* = 0.467; *p* < 0.001), iron-binding capacity (IBC) (*β* = –0.377; *p* < 0.001), and folate (*β* = 0.117; *p* = 0.037) remained independent predictors. Elevated IBC, reflecting functional iron deficiency, was strongly associated with a reduced SDNN, whereas higher folate levels were associated with better autonomic modulation.

**Conclusions:**

In patients presenting with palpitations, the SDNN is influenced by both demographic factors and biochemical markers of iron metabolism. Elevated IBC, reflecting alterations in iron metabolism and iron availability, was associated with impaired autonomic regulation, even in the absence of overt anemia. In contrast, adequate folate status appeared to support a more favorable autonomic function. These findings highlight the importance of integrating iron–vitamin assessment into the evaluation of autonomic function and underscore the need for prospective studies to determine whether correcting these abnormalities can improve HRV and clinical outcomes.

## 1. Introduction

Palpitations, defined as abnormally rapid or irregular heartbeats, may present as skipped beats, fluttering, or a pounding sensation in the chest or neck. Although often benign, they can indicate life-threatening conditions. Palpitations can occur due to structural heart disease or systemic metabolic disorders; however, current knowledge regarding their effects on the autonomic nervous system is limited [1].

Heart rate variability (HRV) is a noninvasive marker of autonomic nervous system function. Reduced HRV is associated with adverse cardiovascular outcomes, including arrhythmias and increased mortality risk [2]. Sex differences in HRV exist, with women showing higher parasympathetic indices and men showing greater sympathetic modulation, indicating sex-specific autonomic regulation [3]. Recent studies have also highlighted autonomic dysfunction in outpatient populations with systemic or post-infectious conditions, emphasizing the clinical relevance of HRV assessment beyond overt cardiovascular disease [4]. Beyond autonomic tone, micronutrient status may also influence HRV. Vitamin B12 and folate are essential cofactors in one-carbon metabolism, and deficiencies can impair hematological and neurological function, potentially altering cardiac autonomic regulation [5]. In functional iron deficiency (FID), inflammation-induced hepcidin upregulation restricts iron availability for erythropoiesis despite adequate or increased iron stores. Unlike absolute iron deficiency, iron-binding capacity (IBC) does not exhibit a compensatory increase and should be interpreted in conjunction with other iron indices and the inflammatory milieu [6]. Combined deficiencies of Vitamin B12 and iron are frequently observed in clinical populations and are associated with distinct hematological and metabolic alterations that may have downstream effects on autonomic regulation [7]. Similarly, it has been shown that HRV is reduced in iron-deficient patients, and parasympathetic nervous system effects are impaired in iron-deficient individuals [8]. The common finding across these studies is that nutritional deficiencies adversely affect autonomic nervous system function and reduce HRV [9, 10, 11, 12]. Recent evidence highlights that sex-specific differences in vitamin metabolism and oxidative stress regulation may contribute to cardiometabolic health disparities, underscoring the importance of integrating nutritional biomarkers into cardiovascular risk assessment [13].

Despite these insights, few studies have simultaneously examined HRV in relation to sex differences and micronutrient status. Understanding these associations may provide novel perspectives on autonomic regulation and its interaction with nutritional deficiencies, particularly in patients with palpitations. Among HRV indices, standard deviation of all normal-to-normal intervals (SDNN) was selected as the primary outcome because it reflects overall autonomic modulation by integrating both sympathetic and parasympathetic influences and is widely accepted as a robust global measure of HRV in clinical and epidemiological studies. This study aimed to investigate sex-specific alterations in HRV and their associations with serum Vitamin B12, folate, and iron levels, thereby contributing to a more comprehensive understanding of cardiovascular risk stratification.

## 2. Methods

### 2.1 Study Design and Ethical Approval

This retrospective observational study was conducted at Konya City Hospital, Türkiye, following approval by the Konya City Hospital Ethics Committee (Approval Date: 10.11.2025; Reference Number: 225/2025). Due to the retrospective design of the study, obtaining informed consent was not considered necessary. All procedures were performed in accordance with the ethical principles of the Declaration of Helsinki and its amendments. The funding bodies had no role in the study design, data collection, interpretation of the results, manuscript preparation, or statistical analyses.

### 2.2 Study Population

A total of 580 adult patients who presented to the cardiology outpatient clinic with palpitations between 2023 and 2024 and underwent 24-hour rhythm Holter monitoring were screened. Patients were identified through the hospital electronic medical record system using the ICD-10 code R00.2 (palpitations). After applying the exclusion criteria, 213 patients aged 18–65 years were included in the final analysis.

### 2.3 Exclusion Criteria

To minimize confounding factors that could influence autonomic nervous system function, patients with the following characteristics were excluded:

● Age <18 or >65 years.

● Known coronary artery disease, heart failure, prior revascularization, and/or coronary surgery.

● Acute coronary syndromes or documented arrhythmias.

● Known autonomic neuropathy.

● Use of any medications known to affect heart rate or autonomic function.

● Acute infectious or inflammatory conditions at the time of Holter monitoring.

● Moderate-to-severe renal impairment (creatinine >2 mg/dL).

● Any chronic inflammatory disease or hematologic malignancy.

● Pregnancy.

### 2.4 Final Cohort

After applying the exclusion criteria, 213 patients (113 women and 100 men) were included in the study cohort. Demographic characteristics, clinical variables, laboratory parameters, and 24-hour Holter-derived HRV indices were extracted from the hospital information system and recorded for statistical analysis.

### 2.5 Biochemical and Laboratory Tests

Biochemical and laboratory data were obtained from the analysis of blood samples collected within the first 24 h of admission. Patients with missing hemogram and biochemistry data were excluded from the study.

### 2.6 24 Hour Ambulatory Rhythm Holter Monitoring 

All participants underwent 24-hour ambulatory electrocardiographic monitoring using the ‘*Promedic digital ECG Holter system*’. Continuous multichannel Electrocardiography (ECG) recordings were obtained during routine daily activities, and the participants were instructed to maintain their usual lifestyle throughout the monitoring period. Holter recordings were analyzed using manufacturer-provided software, which enabled the automated detection of R–R intervals and the calculation of HRV parameters. Automated beat classification was followed by a manual review to ensure the accurate identification of normal-to-normal (NN) intervals. Artifacts, ectopic beats, and noise were excluded from the analysis. Time-domain and frequency-domain HRV parameters were calculated from validated NN interval data in accordance with established international guidelines.

### 2.7 Statistics Analysis

All statistical analyses were performed using the standard procedures for observational cohort studies. Continuous variables are reported as mean ± standard deviation for normally distributed data and as median (interquartile range) for non-normally distributed data. Categorical variables were expressed as frequencies and percentages. The distribution of continuous variables was assessed using the Kolmogorov–Smirnov test. Comparative analyses between female and male participants were performed using the Independent Samples *t*-test for variables exhibiting normal distribution and the Mann–Whitney U test for those not conforming to normal distribution. Categorical variables were compared using Pearson’s chi-squared test or Fisher’s exact test, as appropriate.

Correlations between HRV parameters and iron–vitamin biomarkers (ferritin, serum iron, IBC, folate, and Vitamin B12) were evaluated using Spearman’s rank correlation coefficients because of the non-normal distribution of HRV indices. Correlation analyses were performed for the entire cohort and separately for women and men.

A multivariable linear regression model was constructed to identify the independent predictors of SDNN. Frequency-domain HRV parameters were analyzed in their raw form as provided by the Holter analysis software, without logarithmic transformation. Before multivariable linear regression, multicollinearity among candidate variables was assessed using the variance inflation factor (VIF) analysis, and no significant collinearity was detected. Variables were selected for multivariable modeling based on clinical relevance and their observed associations with SDNN in preliminary and univariate analyses (age, sex, hemoglobin, CRP, ferritin, serum iron, IBC, Vitamin B12, and folate). Standardized beta coefficients (β), 95% confidence intervals (CI), and *p* values were reported. Statistical significance was defined as a two-tailed *p*-value of <0.05.

Statistical analyses were performed using IBM SPSS Statistics (version 27.0; IBM Corp., Armonk, NY, USA) at a significance level of *p* < 0.05.

## 3. Results

### 3.1 Baseline Characteristics

In total, 213 participants (n = 113, 53.1% women and n = 100, 46.9% men) were included in the study. The distributions of the categorical demographic and clinical variables are shown in Table 1. Smoking was significantly more common in men than in women (15.5% vs. 6.6%, *p* < 0.001). Iron deficiency was markedly higher in women than in men (23.0% vs. 1.9%, *p* < 0.001), and folate deficiency was more prevalent among women than among men (5.6% vs. 1.4%, *p* = 0.034). The prevalence of hypertension, diabetes, and thyroid dysfunction did not differ significantly between the sexes. Rhythm Holter findings, including sinus tachycardia, extrasystoles, and paroxysmal atrial fibrillation, were similar between groups.

**Table 1.** **Categorical demographic and clinical characteristics of the study cohort**.

		All Cohort (n = 213)	Women (n = 113, 53.1%)	Men (n = 100, 46.9%)	*p* value
Smoking		47 (22.1)	14 (6.6)	33 (15.5)	< **0.001**
Hypertension		18 (8.4)	9 (4.2)	9 (4.2)	0.81
Diabetes		9 (4.2)	5 (2.3)	4 (1.9)	>0.999
Thyroid Dysfunction		17 (8)	11 (5.2)	6 (2.8)	0.46
	Hypothyroid	16 (7.5)	10 (4.7)	6 (2.8)	-
	Hyperthyroid	1 (0.5)	1 (0.5)	0 (0)	-
Iron Deficiency		53 (24.9)	49 (23)	4 (1.9)	< **0.001**
Vitamin B12 Deficiency		22 (10.3)	15 (7)	7 (3.3)	0.18
Folate Deficiency		15 (7)	12 (5.6)	3 (1.4)	**0.034**
Rhythm Holter Results		213 (100)	113 (53.1)	100 (46.9)	0.68
	Sinus Tachycardia	174 (81.7)	94 (44.1)	80 (37.6)	-
	Atrial or ventricular extrasystole	29 (13.6)	15 (7)	14 (6.6)	-
	Paroxysmal atrial fibrillation	10 (4.7)	4 (1.9)	6 (2.8)	-

Values are n (%). *p* values were calculated using Pearson’s chi-square test or Fisher’s exact test, as appropriate. Significant *p* values are shown in bold.

**Fig. 1.**
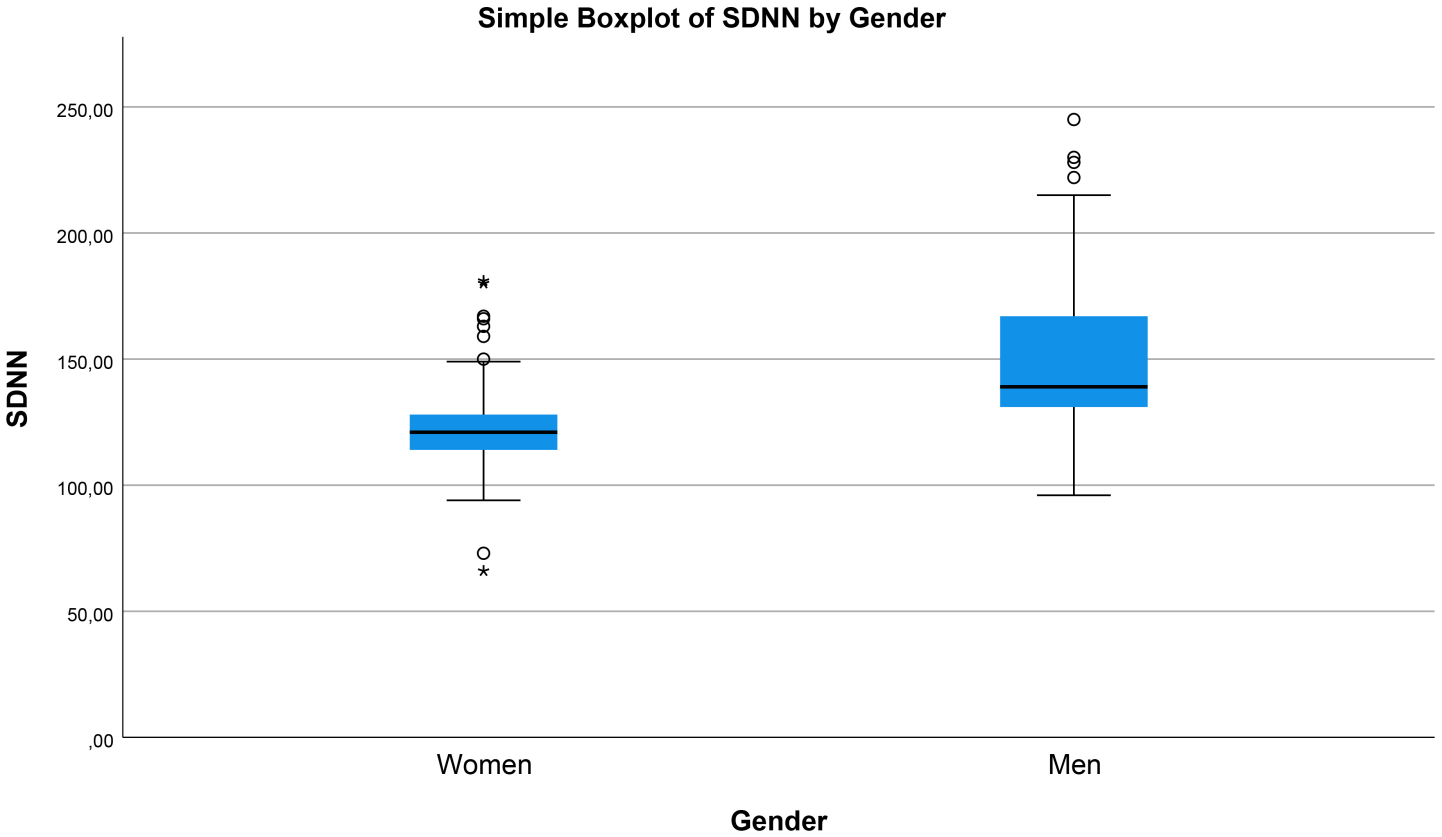
**Box-and-whisker plot of 24-hour SDNN (ms) stratified by sex, showing a higher median SDNN in men than in women**. Outliers are displayed as individual points; overall difference is significant (*p* < 0.001). * and ** indicate outliers. Each is identified by a patient number from the dataset. In the y-axis values, the comma symbol should be interpreted as a decimal point (“.”).

The continuous demographic, biochemical, and HRV parameters are summarized in Table 2. Men had significantly higher levels of creatinine, AST, ALT, ferritin, serum iron, and hemoglobin (all *p* < 0.001), whereas women had higher IBC values (*p* < 0.001). HRV indices, including SDNN, SDNN-INDEX, root Mean Square of Successive Differences (rMSSD), pNN50, and frequency-domain measures, were significantly higher in men than in women (all *p* < 0.001, Fig. 1). Women exhibited higher mean heart rates, whereas men demonstrated lower minimum and higher maximum heart rates.

**Table 2.** **Continuous demographic, biochemical, and Holter-derived parameters**.

	All Cohort (n = 213)	Women (n = 113)	Men (n = 100)	*p* value
Age, years	34.46 ± 12.19	34.14 ± 11.28	34.84 ± 13.19	0.68
GLU, mg/dL	94.63 ± 24.48	93.69 ± 27.2	95.7 ± 21.06	0.55
BUN, mg/dL	11 (9–13)	10 (9–12)	12 (10–13)	<0.001*
CRE, mg/dL	0.71 (0.63–0.84)	0.67 (0.61–0.75)	0.79 (0.69–0.92)	<0.001*
AST, U/L	17 (15–20)	16 (14–19)	19 (15.25–22)	<0.001*
ALT, U/L	17 (12–22)	14 (11–19)	20 (15–29.75)	<0.001*
TSH, mU/L	2.06 ± 1.68	2.21 ± 2.11	1.88 ± 0.99	0.16
CRP, mg/L	1.65 ± 1.2	1.58 ± 1.21	1.74 ± 1.19	0.34
Ferritin, ng/mL	30 (15–66.5)	20 (8–38.5)	50.5 (29–81.75)	<0.001*
Iron, µg/dL	56 (38.5–91.5)	44 (25.5–73)	76.5 (52–101)	<0.001*
IBC, µg/dL	295 (249–367.5)	331 (271–414)	270.5 (235.5–309)	<0.001*
Vitamin B12, pg/mL	288 (244–375)	299 (236.5–417.5)	278 (247.5–345.75)	0.151
Folate, ng/mL	6.69 ± 2.98	6.63 ± 3.28	6.74 ± 2.62	0.78
WBC, ×10^3^/µL	7.67 ± 1.62	7.63 ± 1.75	7.71 ± 1.46	0.72
HB, g/dL	13.7 (12.65–15.3)	12.8 (12–13.65)	15.2 (13.9–16.4)	<0.001*
Mean_HR, bpm	79 (73–84)	81 (77–85)	77 (69–80.75)	<0.001*
Lowest_HR, bpm	50 (45–54)	52 (48–56)	46 (43–51)	<0.001*
Highest_HR, bpm	142.5 ± 16.5	145.9 ± 16.01	138.67 ± 16.27	0.001**
SDNN (24 h), ms	129 (119–144)	121 (114–128)	139 (131–167)	<0.001*
SDNN-INDEX (24 h), ms	50 (41–62)	44 (37–49)	59 (51.25–75)	<0.001*
rMSSD, ms	31 (26–36)	29 (25–34)	32 (26.25–44)	<0.001*
pNN50, (%)	14 (11–18)	14 (9–16.5)	15 (12–21)	<0.001*
TRIANGULAR INDEX (24 h)	22 (17.5–28)	18 (16–23)	26 (22–32.75)	<0.001*
HF, ms^2^	486.9 (419.25–650.25)	573.4 (394.65–650.25)	467.45 (42.66–648.35)	0.762
LF, ms^2^	632.1 (496.45–716.8)	541.9 (463.6–646.8)	686.45 (628.65–906.93)	<0.001*
VLF, ms^2^	1166.9 (773.05–1534.55)	871.2 (673.65–1082.8)	1532.7 (1426.1–1609.58)	<0.001*
LF/HF	1.21 (0.98–1.52)	1.04 (0.94–1.19)	1.46 (1.25–1.64)	<0.001*

Continuous variables are reported as mean ± standard deviation for normally distributed data and as median (interquartile range) for non‑normally distributed data. *Significant at *p* < 0.05 level, the Mann-Whitney U test. **Significant at *p* < 0.05 level, Independent Samples *T*-Test. GLU, Glucose; BUN, Blood Urea Nitrogen; CRE, Creatinine; AST, Aspartate aminotransferase; ALT, Alanine aminotransferase; TSH, Thyroid-Stimulating Hormone; CRP, C-Reactive Protein; IBC, Iron-Binding Capacity; WBC, White Blood Cell Count; HB, Hemoglobin; Mean_HR, Mean Heart Rate; SDNN, Standard Deviation of all normal-to-normal Intervals; SDNN-INDEX, Mean of the Standard Deviations of NN intervals for all 5-minute segments; rMSSD, root Mean Square of Successive Differences; pNN50, Percentage of successive NN Intervals differing by more than 50 ms; HF, High-Frequency; LF, Low-Frequency; VLF, Very-Low-Frequency; LF/HF, Low-to-High Frequency.

### 3.2 Correlations Between HRV Parameters and Iron–Vitamin Biomarkers

The Spearman correlation coefficients for the entire cohort are presented in Table 3. All major time-domain HRV parameters (SDNN, SDNN-INDEX, rMSSD, pNN50, TRIANGULAR INDEX) showed significant correlations with ferritin, serum iron, IBC, folate, and Vitamin B12 (*p* < 0.05). The Low-to-High Frequency (LF/HF) Ratio demonstrated weaker but still significant correlations with ferritin, iron, and IBC, whereas its associations with folate and Vitamin B12 were not statistically significant.

**Table 3.** **Spearman correlation coefficients (ρ) between HRV parameters and iron–vitamin status in the whole cohort**.

HRV Parameters	Ferritin (ρ)	Iron (ρ)	IBC (ρ)	Folate (ρ)	Vit. B12 (ρ)
SDNN (24 h), ms	0.314**	0.272**	0.210**	0.162*	0.148*
SDNN-INDEX (24 h), ms	0.291**	0.249**	0.192**	0.158*	0.139*
rMSSD, ms	0.278**	0.244**	0.189**	0.151*	0.132*
pNN50, (%)	0.265**	0.238**	0.183**	0.146*	0.128*
TRIANGULAR INDEX (24 h)	0.301**	0.263**	0.203**	0.161*	0.142*
LF/HF	0.198**	0.174*	0.142*	0.119	0.103

Positive correlation coefficients indicate higher HRV values with higher micronutrient levels. Spearman’s rank correlation coefficient (ρ). *p < 0.05, **p < 0.01. HRV, heart rate variability.

Sex-stratified-analyses revealed distinct patterns. Notably, correlation coefficients between HRV parameters and iron–vitamin biomarkers were approximately two-fold higher in women than in men, indicating a stronger association between micronutrient status and autonomic modulation in female participants (Tables 4,5). In men, the correlations between HRV indices and iron–vitamin biomarkers were modest, reaching significance mainly for ferritin and serum iron. In contrast, women exhibited consistently stronger correlations across all HRV parameters, particularly with ferritin, iron, IBC, folate, and Vitamin B12 levels (all *p* < 0.01). These findings suggest a more pronounced sensitivity of autonomic function to micronutrient status in females.

**Table 4.** **Spearman correlation coefficients (ρ) between HRV parameters and iron–vitamin status in Men**.

HRV Parameters	Ferritin (ρ)	Iron (ρ)	IBC (ρ)	Folate (ρ)	Vit. B12 (ρ)
SDNN (24 h), ms	0.228*	0.204*	0.171	0.148	0.132
SDNN-INDEX (24 h), ms	0.213*	0.192	0.163	0.139	0.127
rMSSD, ms	0.205*	0.187	0.158	0.135	0.121
pNN50, (%)	0.198*	0.179	0.152	0.129	0.116
TRIANGULAR INDEX (24 h)	0.219*	0.196	0.167	0.142	0.129
LF/HF	0.162	0.148	0.131	0.118	0.104

Positive correlation coefficients indicate higher HRV values with higher micronutrient levels. Spearman’s rank correlation coefficient (ρ). *p < 0.05.

**Table 5.** **Spearman correlation coefficients (ρ) between HRV parameters and iron–vitamin status in Women**.

HRV Parameters	Ferritin (ρ)	Iron (ρ)	IBC (ρ)	Folate (ρ)	Vit. B12 (ρ)
SDNN (24 h), ms	0.412**	0.379**	0.296**	0.241*	0.218*
SDNN-INDEX (24 h), ms	0.398**	0.361**	0.284**	0.229*	0.204*
rMSSD, ms	0.385**	0.347**	0.271**	0.218*	0.196*
pNN50, (%)	0.371**	0.336**	0.263**	0.211*	0.189*
TRIANGULAR INDEX (24 h)	0.403**	0.368**	0.287**	0.236*	0.213*
LF/HF	0.241*	0.218*	0.179	0.152	0.138

Positive correlation coefficients indicate higher HRV values with higher micronutrient levels. Spearman’s rank correlation coefficient (ρ). *p < 0.05, **p < 0.01.

### 3.3 Univariate and Multivariate Linear Regression Analysis for SDNN

Univariate regression analyses demonstrated that age was negatively associated with SDNN (β = –0.203, *p* = 0.003), whereas male sex was strongly associated with higher SDNN (β = 0.529, *p* < 0.001). These variables, along with clinically relevant biochemical markers, were included in the multivariate model. The multivariable regression model explained 42.7% of the variance in SDNN (R^2^ = 0.427). The multivariate linear regression model predicting the SDNN is shown in Table 6. Age was independently associated with a lower SDNN (β = –0.269, *p* < 0.001), whereas male sex was a strong positive predictor (β = 0.467, *p* < 0.001). IBC was significantly negatively associated with SDNN (β = –0.377, *p* < 0.001), indicating that a higher IBC was related to reduced HRV. Folate level was an independent positive predictor (β = 0.117, *p* = 0.037). Ferritin, serum iron, Vitamin B12, hemoglobin, and CRP levels were not significant in the adjusted model.

**Table 6.** **Univariate and multivariable linear regression analyses for predictors of SDNN**.

	Univariate Analysis		Multivariate Analysis			Interpretation
	Standardized Coefficients	*p*-value	Standardized Coefficients	95.0% Confidence Interval for B	*p*-value	
	Beta (ß)		Beta (ß)	[95% CI]		
Age	–0.203	0.003*	−0.269	−0.88 to −0.34	<0.001*	SDNN decreases with age
Gender	0.529	<0.001*	0.467	18.5 to 33.5	<0.001*	SDNN is higher in men
HB, g/dL	0.40	<0.001*	−0.052	−3.11 to 1.55	0.51	Not significant
CRP, mg/L	–0.088	0.202	−0.035	−3.39 to 1.77	0.53	Not significant
Ferritin, ng/mL	0.313	<0.001*	−0.048	−0.12 to 0.06	0.50	Not significant
Iron, µg/dL	0.345	<0.001*	−0.074	−0.15 to 0.05	0.34	Not significant
IBC, µg/dL	–0.448	<0.001*	−0.377	−0.18 to −0.06	<0.001*	Higher IBC → lower SDNN
Vitamin B12, pg/mL	0.052	0.448	0.056	−0.01 to 0.02	0.30	Not significant
Folate, ng/mL	0.097	0.159	0.117	0.06 to 2.11	0.037*	Higher folate → higher SDNN

*Significant at *p* < 0.05 level, R^2^ = 0.427.

## 4. Discussion

In the present study, we observed marked sex-specific differences in HRV parameters, with women demonstrating significantly lower time- and frequency-domain indices (SDNN, rMSSD, pNN50, Low-Frequency (LF) Power, and Very-Low-Frequency (VLF) Power) than men. This is consistent with prior observations of sex differences in autonomic cardiac regulation, in which men typically exhibit higher overall HRV, reflecting greater autonomic flexibility [3, 14]. Multivariable regression analysis further confirmed that male sex was independently associated with higher SDNN (β = 0.467, *p* < 0.001, Fig. 1), whereas SDNN decreased with advancing age (β = –0.269, *p* < 0.001). Umetani *et al*. [15] reported a progressive age-related decline in multiple time-domain HRV measures, including SDNN, across nine decades in healthy subjects, underscoring the physiological reduction in autonomic modulation with aging.

Iron deficiency was significantly more prevalent in women (23% vs. 1.9%, *p* < 0.001), accompanied by lower ferritin, serum iron, and hemoglobin levels and higher IBC. Importantly, IBC may capture early or subclinical disturbances in iron availability that are not fully reflected by ferritin levels alone, thereby providing additional insight into iron-related autonomic dysregulation even in the absence of overt anemia. Spearman’s correlation revealed positive associations between HRV parameters and iron markers (ferritin and serum iron), which were notably stronger in women. These findings align with evidence linking iron deficiency anemia to impaired autonomic function, primarily through reduced parasympathetic activity and sympathetic predominance [8, 16]. For example, Tuncer *et al*. [16] demonstrated lower HRV in patients with iron deficiency anemia than in controls, attributing this to hypoxia-induced sympathetic activation. Similarly, in patients with coronary heart disease or chronic anemia (e.g., thalassemia), anemia is associated with diminished HRV, potentially mediating a heightened cardiovascular risk [17, 18]. Higher IBC emerged as an independent negative predictor of SDNN (β = –0.377, *p* < 0.001), further supporting the role of latent iron deficiency in the autonomic dysregulation. Although some correlations were statistically significant, the magnitude of the other relationships was modest, and some of the findings should be interpreted as weak clinical correlations rather than strong relationships. The stronger correlations observed in women may partly reflect menstrual blood loss and hormonal influences on iron homeostasis, exacerbating their vulnerability to autonomic dysfunction. Elevated IBC may reflect reduced bioavailable iron, potentially impairing oxygen transport, mitochondrial oxidative phosphorylation, and cellular energy production. These alterations may activate compensatory neurohumoral pathways and sympathetic drive, thereby contributing to autonomic imbalance and reduced HRV [19].

Likewise, folate deficiency was more common in women (5.6% vs. 1.4%, *p* = 0.034), with positive correlations between HRV indices and folate levels, which were stronger in females than in males. Folate independently predicted higher SDNN in the regression analysis (β = 0.117, *p* = 0.037). Vitamin B12 deficiency was associated with weaker but positive correlations with HRV. These nutrient-HRV links are supported by studies indicating that Vitamin B12 and folate deficiencies disrupt autonomic balance, possibly via hyperhomocysteinemia, oxidative stress, or impaired neuronal myelination [11, 12]. Sucharita *et al*. [20] found reduced HRV in Vitamin B12-deficient patients, reversible with replacement therapy. Supplementation trials have similarly shown improvements in HRV parameters following B12 repletion in elderly participants. Folate deficiency is associated with impaired nitric oxide bioavailability, endothelial dysfunction, and increased sympathetic drive. Although direct evidence for the effect of folate supplementation on HRV is limited, its role in homocysteine metabolism suggests that it shares pathways with B12 in maintaining the vagal tone [21, 22].

Mechanistically, these micronutrients contribute to erythropoiesis, redox balance, and neurotransmitter synthesis, and deficiencies may promote sympathetic dominance, as evidenced by higher LF/HF ratios in deficient states. Sex disparities likely stem from reproductive factors that increase the deficiency risk in women, amplifying the autonomic effects [19]. Despite the non-significant association with CRP, subclinical inflammation cannot be fully excluded.

## 5. Limitations

This study has several limitations related to its design, patient population, and single-center nature.

● Its retrospective design precludes causal inference, and unmeasured confounders may still be present despite the strict exclusion criteria.

● Although ferritin, iron, and inflammatory markers were evaluated, ferritin can be influenced by subclinical inflammation, and more sensitive inflammatory biomarkers (e.g., IL-6 and hepcidin) were not available in the dataset used.

● In addition, detailed information on dietary intake and micronutrient supplementation was not available, which may have influenced circulating iron, folate, and Vitamin B12 levels.

● HRV parameters were derived from 24-hour Holter recordings, which provide a robust assessment of autonomic function but may still be influenced by daily activity patterns, sleep quality, and psychological stress, factors that cannot be fully standardized or quantified retrospectively.

● The study population consisted of individuals presenting with palpitations, which may limit the generalizability of the results to asymptomatic or community-based populations. The study population may not be representative of the general population, limiting the generalizability of the findings.

● Finally, although the sample size was adequate for multivariable modeling, the inclusion of additional biochemical markers or longitudinal follow-up could further strengthen the mechanistic interpretation of the findings.

## 6. Conclusions

In this cohort of adults presenting with palpitations, SDNN, a key marker of global autonomic modulation, was strongly influenced by age, sex, and biochemical indices of iron metabolism. Male sex and younger age were associated with higher SDNN values, underscoring the importance of demographic stratification when interpreting HRV metrics in clinical settings. Although ferritin, serum iron, folate, and Vitamin B12 showed significant correlations with HRV parameters, only IBC and folate remained independent predictors in the multivariate analysis. These findings suggest that alterations in iron metabolism, reflected by elevated IBC, may impair autonomic regulation even in the absence of overt anemia, whereas adequate folate status may support healthier autonomic function.

Overall, our results highlight the need to consider demographic, biochemical, and nutritional factors when evaluating HRV in patients with palpitations. Identifying modifiable biochemical factors that influence HRV may offer opportunities for early intervention in patients with palpitations. Moreover, the strong effects of age and sex emphasize the need for demographic adjustments when interpreting HRV metrics in clinical settings. Given the retrospective observational design, the associations observed in this study do not imply causality. Therefore, no conclusions can be drawn regarding the potential benefits of iron or folate supplementation, which should be evaluated in future prospective and interventional studies. Future prospective studies are warranted to clarify the mechanistic pathways linking iron homeostasis and autonomic balance and to determine whether correcting iron handling-abnormalities or folate deficiency can improve HRV and clinical outcomes.

## Data Availability

The data used in this study are available upon reasonable requests.

## Abbreviations

ALT, Alanine aminotransferase; AST, Aspartate aminotransferase; BUN, Blood Urea Nitrogen; CRE, Creatinine; CRP, C-Reactive Protein; ECG, Electrocardiography; GLU, Glucose; HB, Hemoglobin; HF, High-Frequency; HR, Heart Rate; HRV, Heart Rate Variability; IBC, Iron-Binding Capacity; LF, Low-Frequency; LF/HF, Low-to-High Frequency; NN, Normal-to-Normal Intervals; Mean_HR, Mean Heart Rate; pNN50, Percentage of successive NN Intervals differing by more than 50 ms; rMSSD, root Mean Square of Successive Differences; SDNN, Standard Deviation of all NN Intervals; SDNN-index, Mean of the Standard Deviations of NN intervals for all 5-minute segments; TSH, Thyroid-Stimulating Hormone; VLF, Very-Low-Frequency; WBC, White Blood Cell Count.
